# Expert Consensus on the Long-Term Effectiveness of Medical Nutrition Therapy and Its Impact on the Outcomes of Adults with Phenylketonuria

**DOI:** 10.3390/nu15183940

**Published:** 2023-09-11

**Authors:** Júlio César Rocha, Kirsten K. Ahring, Heather Bausell, Deborah A. Bilder, Cary O. Harding, Anita Inwood, Nicola Longo, Ania C. Muntau, André L. Santos Pessoa, Fran Rohr, Serap Sivri, Álvaro Hermida

**Affiliations:** 1 NOVA Medical School, Faculdade de Ciências Médicas, Universidade NOVA de Lisboa, Campo Mártires da Pátria 130, 1169-056 Lisboa, Portugal; 2 Reference Centre of Inherited Metabolic Diseases, Centro Hospitalar Universitário de Lisboa Central, Rua Jacinta Marto, 1169-045 Lisboa, Portugal; 3 CINTESIS@RISE, Nutrition and Metabolism, NOVA Medical School, Faculdade de Ciências Médicas, NMS, FCM, Universidade NOVA de Lisboa, Campo Mártires da Pátria 130, 1169-056 Lisboa, Portugal; 4 Departments of Paediatrics and Clinical Genetics, PKU Clinic, Copenhagen University Hospital, Rigshospitalet, Blegdamsvej 9, 2100 Copenhagen, Denmark; 5 Division of Genetics, Genomics, and Metabolism, Ann & Robert H Lurie Children’s Hospital of Chicago, 225 E. Chicago Ave., Chicago, IL 60611, USA; 6 Department of Psychiatry, Division of Child & Adolescent Psychiatry, University of Utah Huntsman Mental Health Institute, 501 Chipeta Way, Salt Lake City, UT 84108, USA; 7 Department of Molecular and Medical Genetics, Oregon Health & Science University, 3222 SW Research Drive, Portland, OR 97239, USA; 8 Queensland Lifespan Metabolic Medicine Service, Queensland Children’s Hospital, 501 Stanley St., South Brisbane, QLD 4101, Australia; 9 School of Nursing and Social Work, The University of Queensland, Chamberlain Building, St. Lucia, QLD 4072, Australia; 10 Department of Pediatrics, Division of Medical Genetics, University of Utah School of Medicine, 295 Chipeta Way, Salt Lake City, UT 84108, USA; 11 Department of Pediatrics, University Children’s Hospital, University Medical Centre Hamburg-Eppendorf, Martinistraße 52, 20246 Hamburg, Germany; 12 Albert Sabin Children’s Hospital, R. Tertuliano Sales, 544—Vila União, Fortaleza 60410-794, CE, Brazil; 13 Av. Dr. Silas Munguba, 1700—Itaperi, State University of Ceará (UECE), Fortaleza 60714-903, CE, Brazil; 14 Met Ed, Boulder, CO 80302, USA; 15 Division of Pediatric Metabolism, Department of Pediatrics, Faculty of Medicine, Hacettepe University, Gevher Nesibe Cd., 06230 Ankara, Turkey; 16 Diagnosis and Treatment of Congenital Metabolic Diseases Unit (UDyTEMC), Department of Pediatrics, Faculty of Medicine, Clinical University Hospital of Santiago de Compostela, University of Santiago de Compostela, CIBERER, MetabERN, Institute of Clinical Research of Santiago de Compostela (IDIS), Rúa de San Francisco s/n, 15706 Santiago de Compostela, Spain

**Keywords:** phenylketonuria, PKU, adult, comorbidities, medical nutrition therapy, low Phe diet, modified Delphi, consensus recommendations

## Abstract

Many adults with phenylketonuria (PKU) rely on medical nutrition therapy (MNT; low phenylalanine (Phe) diet with protein substitutes/medical foods) to maintain blood Phe concentrations within recommended ranges and prevent PKU-associated comorbidities. Despite disease detection through newborn screening and introduction of MNT as early as birth, adherence to MNT often deteriorates from childhood onwards, complicating the assessment of its effectiveness in the long term. Via a modified Delphi process, consensus (≥70% agreement) was sought on 19 statements among an international, multidisciplinary 13-member expert panel. After three iterative voting rounds, the panel achieved consensus on 17 statements related to the limitations of the long-term effectiveness of MNT (7), the burden of long-term reliance on MNT (4), and its potential long-term detrimental health effects (6). According to the expert panel, the effectiveness of MNT is limited in the long term, is associated with a high treatment burden, and demonstrates that adults with PKU are often unable to achieve metabolic control through dietary management alone, creating an unmet need in the adult PKU population.

## 1. Introduction

Phenylketonuria (PKU) results from biallelic pathogenic variants in the phenylalanine hydroxylase (*PAH*) gene and is identified in medically advanced countries via newborn screening detection of elevated blood phenylalanine (Phe) concentrations [1,2]. Immediately after diagnosis, medical nutrition therapy (MNT) is initiated to reduce blood Phe concentrations, preventing the development of severe and often irreversible neurological, behavioural and psychiatric symptoms [1,2,3]. The components of MNT for PKU include a diet restricted in Phe with the intake of protein substitutes/medical foods. This often means complete avoidance of the intake of high-protein foods, such animal and dairy products, nuts and seeds, legumes and soya. As a result, patients rely on foods with a naturally low protein content (e.g., fruits and vegetables) and modified low-protein foods from specialised manufacturers (e.g., bread and pasta) to meet their energy requirements [4,5]. To replace the intake of natural protein, Phe-free protein substitutes or medical foods are an integral part of MNT for preventing protein deficiency and ensuring normal growth among other health outcomes [4,5]. In addition to MNT, sapropterin dihydrochloride, a synthetic analogue of tetrahydrobiopterin (BH_4_), the natural cofactor of the PAH enzyme, can be offered to a portion of the PKU population expressing residual PAH enzymatic activity [1,2,6]. However, about 50–80% of patients, especially those with a more severe disease phenotype, are unresponsive to sapropterin [7]. Pegvaliase (PALYNZIQ^®^) can be an option for adults with uncontrolled blood Phe (>600 µmol/L) in the United States (US) or for those >16 years of age in Europe [8,9].

Because these pharmacological treatments are not universally available or an option for all patients, a substantial part of the adult PKU population rely on MNT alone for management of their disease. However, adherence to MNT is a challenge and known to decrease with age. Studies have reported that approximately 50% of the adolescent and adult PKU population cannot maintain blood Phe concentrations within the European guideline-recommended target range (120–600 µmol/L) [10,11]. The number of patients who are unsuccessful in attaining guideline-recommended target ranges further increases when considering only those with a severe disease phenotype or when applying the American College of Medical Genetics (ACMG) target range (120–360 µmol/L) [10,11,12,13,14]. The lack of lifelong blood Phe control in patients on MNT puts them at risk for developing cognitive and executive function deficits and other psychological and neurologic abnormalities, although evidence on these symptoms in the adult PKU population is mostly limited to smaller observational studies and case series [15]. In addition, studies on somatic comorbidities in PKU adults are scarce, and the results are often conflicting [16,17].

Due to the paucity of evidence on the long-term outcomes of adults with adequately controlled PKU, expert opinions were collected through a modified Delphi process, leading to the development of a set of consensus statements that aim to shed light on the long-term effectiveness of MNT and improve the understanding of the impact of MNT on the health outcomes of adults with PKU.

## 2. Materials and Methods

**Figure 1:**
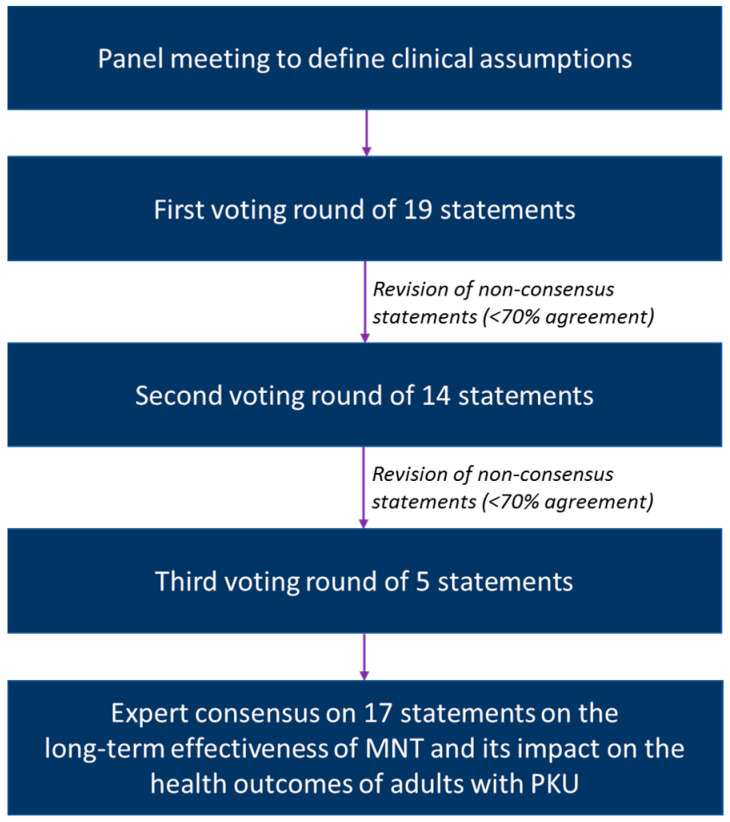
Overview of the modified Delphi process.

A modified Delphi process was applied to gain consensus from an international, multidisciplinary 13-member panel of experts with extensive experience in the management of adults with PKU (Figure 1). The panel consisted of four metabolic physicians, four dietitians, two specialists in internal medicine, one neurologist, one nurse practitioner and one psychiatrist. Different management perspectives were represented by the geographic diversity of the group. Based on a virtual expert panel meeting organised in January 2022, 19 statements were developed across three topics:

- Limitations of long-term effectiveness of MNT (7 statements);
- Burden of long-term reliance on MNT (4 statements);
- Potential detrimental health effects related to long-term reliance on MNT (8 statements).

Through an iterative process of individual voting rounds on a secure online platform, each of these statements was assessed by the expert panel along with providing feedback when not accepting a given statement. The gathered feedback was used to revise the statements without consensus (<70% agreement), which were voted on again in two subsequent rounds. All statements included the option “can’t judge” in case the expert lacked experience to vote on a specific statement. If the option “can’t judge” was chosen, the answer was excluded from the agreement calculations.

## 3. Results

### 3.1. Limitations of Long-Term Effectiveness of MNT


**Statement #1**

**Consensus**
Due to a combination of factors, the sustainability of MNT worsens from early adolescence onwards to an extent that strictly following the Phe-restricted diet becomes difficult for most patients, limiting the effectiveness of MNT to control blood Phe concentrations in the long term.100%

Studies in both Europe and the US have demonstrated that achievement of guideline-recommended target ranges starts to worsen during early adolescence and declines further in adulthood [10,11,12,13,18,19,20,21], while not even accounting for the high number of patients lost to follow-up of whom many are expected to live with poor metabolic control [22,23]. The deterioration of metabolic control is especially problematic for BH4-unresponsive patients with a severe disease phenotype who have the lowest dietary Phe tolerance [10]. The reasons for the limited sustainability of MNT are multifactorial and extend beyond the difficulties of simply avoiding foods containing Phe [24]. In addition to the burden of restricting dietary Phe, the consumption of protein substitutes/medical foods can interfere significantly with activities of daily life (e.g., work, educational activities, sports, travelling and dining out) and lead to dietary stigma and feelings of social exclusion [24,25,26,27,28]. Other obstacles limiting adherence in the long term include the financial cost and time burden associated with the daily intake of protein substitutes/medical foods and the lack of palatability, as described further in the topic on the burden of long-term reliance on MNT [24,28,29].
**Statement #2****Consensus**For adults with PKU on MNT, the ability to maintain blood Phe concentrations ≤360 µmol/L throughout life is limited to a minority of patients having a milder disease phenotype, significant discipline in the setting of adequate resources and continued access to care, or to temporary circumstances of intensive medical involvement and support, such as pregnancy.100%

It has been reported in the literature that patients adherent to MNT do not always succeed in achieving metabolic control [30,31]. Continuous access to care through paediatric-to-adult-care transition programmes can to some extent prevent loss to follow-up and the deterioration of blood Phe control in adulthood [31,32]. However, these programmes are available in only a few centres, with a general lack of access to adult services remaining for many patients [33]. It was the opinion of the expert panel that for some patients, blood Phe concentrations can only be controlled under conditions of intensive medical involvement and support. Prevention of maternal PKU syndrome has proven to be one of these conditions, as it is an important incentive for female patients to temporarily reduce blood Phe concentrations, but even still, about 30% of pregnant women struggle to achieve blood Phe concentrations below 360 µmol/L despite the implementation of dietary management [11,34]. Based on the experiences of the expert panel, part of the adult PKU population will have extreme difficulty achieving metabolic control by adhering to a Phe-restricted diet unless significant clinical support is provided, which may include hospitalisation in the case of maternal PKU. However, such intensive support can only be offered temporarily and has downstream effects on a patient’s quality of life (QoL) and other health parameters.
**Statement #3****Consensus**The majority of adults with PKU are unable to reach and sustain physiological blood Phe concentrations by strictly adhering to MNT, including a medically prescribed Phe-restricted diet.100%**Statement #4****Consensus**Current treatment targets, albeit evidence-based, allow blood Phe concentrations that are above the physiological range due to the lack of data on the outcomes of adults with PKU with physiological blood Phe concentrations (30–120 µmol/L).100%

Recent retrospective observational studies as well as expert opinions have led to the conclusion that it is nearly impossible to achieve physiological blood Phe concentrations (30–120 µmol/L) on MNT, even in adults who strictly follow their dietary restrictions [10,11,31]. In fact, to date, no studies have been published that examined the outcomes of MNT-treated PKU adults with physiological blood Phe irrespective of the disease severity. Recognising that physiologically normal blood Phe is generally not achievable with MNT, treatment guidelines for PKU allow concentrations of the disordered metabolite that are at least three times as high as the normal range. Furthermore, it should be noted that the lower treatment target (120 µmol/L) is historical and based on the limited sensitivity of the once widely employed Guthrie bacterial inhibition assay (1), with actual reference blood Phe concentrations in healthy adults being as low as 35–85 µmol/L [35].
**Statement #5****Consensus**The current treatment targets may not fully prevent the risk for developing PKU-associated comorbidities, including difficulties in neurocognitive functioning.100%**Statement #6****Consensus**Adults with PKU are prone to develop deficits in executive functioning, impeding their ability to manage the complexities of their diet, including intake of low Phe foods and frequent administration of protein substitutes/medical foods.77%

While currently available and emerging pharmacological treatment options (including pegvaliase) may allow patients to achieve and sustain physiological blood Phe concentrations [36], the significance of this achievement as it relates to patient health and outcomes is still to be determined. Until then, the potential benefits of achieving and maintaining physiological blood Phe can only be evaluated in studies comparing adults with PKU to unaffected controls. These comparative studies have shown that early-treated adults with PKU generally have normal cognition if they maintain blood Phe below 360 µmol/L throughout childhood, although subtle deficits remain in some adult patients due to early diet relaxation [37,38,39,40,41,42,43,44,45]. As most studies failed to include a population of adults with PKU with mean blood Phe concentrations below 600 µmol/L, there remains much uncertainty whether current treatment targets are also safe in adulthood. A single study compared the neurocognitive outcomes of early-treated adults with PKU with blood Phe concentrations above and below 600 µmol/L with those of healthy controls [46]. Regardless of blood Phe, patients with PKU had lower performance scores on tests of memory, problem-solving skills and strategy than healthy controls, arguing that an upper blood Phe target of 600 µmol/L may not be strict enough for the entire adult PKU population. However, opposite conclusions have been published elsewhere [39] and hence, the safety of treatment targets in PKU will remain a matter of debate until a larger portion of adults with PKU are able to achieve and maintain blood Phe concentrations below at least 600 µmol/L with available treatment options. Despite disagreement on the safety of the upper blood Phe target, it is clear that even in adulthood, blood Phe concentrations above 600 µmol/L are detrimental to mental health outcomes (e.g., anxiety) and executive function, complicating the planning of low-protein meals and impacting the ability to schedule in-clinic appointments, which for some leads to a cycle of suboptimal treatment adherence [15,26,41,47,48].
**Statement #7****Consensus**Due to the sustainability challenges related to MNT, adults with PKU should be offered pharmacological treatments to lower blood Phe levels when available.85%

According to a recent survey, about 70% of adult patients are dissatisfied with treatment plans that still require protein restriction [49]. For some of them, pegvaliase can be considered. However, pegvaliase is not yet available in all countries, and the benefits of substantial blood Phe reductions are often preceded by hypersensitivity-related adverse events, especially during the first months of treatment [36,49,50,51,52,53]. Nevertheless, patients report a willingness to accept the possibility of side effects in exchange for lower blood Phe concentrations that can be within the physiological range [49]. In addition, pegvaliase allows most patients to consume a more normal diet, decreasing the need for protein restriction but requiring further guidance in order to maintain a balanced nutritional intake to prevent imbalances and avoid periods of hypophenylalaninemia [54,55,56]. Although suboptimal adherence to previous treatments should not exclude patients from receiving pegvaliase, hypersensitivity reactions, injection fatigue and the variable time to response may lead to discontinuation in a minority of patients who commence therapy [57,58,59,60]. For patients responsive to BH4, the addition of sapropterin to the Phe-restricted diet remains a valuable treatment option. Adherence to sapropterin was reported to be dependent on the extent of blood Phe reduction along with the increase in Phe tolerance, furthermore improving impairments in attention and executive functions [6,54,61,62,63,64,65,66,67,68]. However, for most patients on sapropterin, diet can only be partially normalised, which does not completely alleviate the burden of MNT [69,70]. Despite the progress made, researchers continue to search for new treatment modalities that can further minimise the burden of PKU management and ultimately address the unmet treatment need in the long term [71].

### 3.2. Burden of Long-Term Reliance on MNT


**Statement #8**

**Consensus**
In some countries, the availability and reimbursement of prescribed special low-protein foods and protein substitutes/medical foods are limited, resulting in a high financial and time burden that can cause worry and stress in the adult PKU population.100%
**Statement #9**

**Consensus**
In adults with PKU, poor metabolic control can contribute to mental health and psychosocial issues. These issues can be exacerbated by the burden of MNT, especially in countries with limited access to protein substitutes/medical foods and inadequate dietetic support.100%

Limited adherence of adult patients to MNT is not always by choice. In many regions, reimbursement of special manufactured low-protein foods and protein substitutes/medical foods remains an issue, especially for the adult PKU population [14,72,73,74,75,76,77,78,79,80]. Suboptimal access to dietary products generates inequality and high out-of-pocket costs, causing a financial burden that can discourage patients from staying adherent to MNT [81,82]. Even in countries such as the United Kingdom (UK), where special manufactured low-protein foods and protein substitutes are fully covered, maintaining a continuous supply of dietary products can be problematic [83]. When dietary products are insufficiently available, the ability to control blood Phe is beyond the willingness of the patient, increasing the risk of impaired neurocognitive functioning that can affect employment and socioeconomic status and further limit access to care [14,84]. In addition to the financial burden, the implementation of MNT into daily life is complex and time-consuming, particularly for patients who require the most severe dietary protein restrictions [24,26,27,72,85]. Based on studies in the Netherlands and the US, adults with PKU spend approximately 30–50 min per day on PKU-related tasks, including the preparation of special meals and monitoring of protein intake [72,85]. This time burden is inherent to MNT and further worsens if access to dietary products cannot be guaranteed, an issue that has been described in the literature and by the experts to occur not only in the UK but also in other countries, such as Spain, Turkey and the US [83]. Hence, lifelong adherence to MNT can be stressful to adults with PKU who already have a higher risk of developing mental-health-related and psychosocial issues than the general population, especially in regions where access to care and quality of nutrition support are limited [15,25,26,37,42,83,86,87,88]. Though availability of pharmacological treatments currently remains an issue in some countries, they may reduce the out-of-pocket costs and time burden associated with adherence to MNT if they allow diet normalisation, while blood Phe reduction may further improve mental health and other PKU-related comorbidities [6,89,90].
**Statement #10****Consensus**Elevated blood Phe concentrations increase the risk for the development of neuropsychological comorbidities, such as anxiety and depression. These can be exacerbated in patients with attempted but unsuccessful adherence to MNT, who often experience feelings of guilt and decreased emotional well-being.85%

There is consensus that patients with PKU should avoid elevations in blood Phe throughout life to preserve neurotransmitter production and myelin synthesis [1,2,3]. Although the neurotoxic effects of Phe seem to vary between individuals, there is a general trend of increased vulnerability to mental health disorders, including mood disturbances, anxiety and depression, correlated with blood Phe concentrations in adulthood [15,21,42,48,91,92,93,94]. However, in some studies, the relationship between biochemical markers and neuropsychological symptoms was not significant [95,96,97]. In addition to blood Phe, the burden of living with a chronic and rare metabolic condition requiring lifelong management and routine follow-up can contribute to the development of internalised problems (i.e., symptoms that are directed inward and experienced within the individual) that can be addressed by involving mental health providers in PKU care [98,99,100]. Similarities in terms of depressive mood, anxiety and social isolation have been reported between PKU and other chronic metabolic conditions, including diabetes [99]. Compared with diabetic patients who can easily self-monitor their insulin levels, patients with PKU rely on the results of submitted blood spots that can take 1–2 weeks to receive, contributing to the treatment burden. Currently, home Phe-monitoring systems are still in development [101]. When available, such a system may increase patient self-empowerment regardless of therapeutic management [100,101].

Studies have shown that in particular, patients with PKU whose attempts to strictly follow the dietary restrictions are unsuccessful perceive their treatment burden to be high, which, for some, can cause guilt and embarrassment [37,47,81,88]. Although the emotional well-being of patients who are able to successfully integrate MNT was reported to be higher compared with patients who experience adherence difficulties, they may find it more difficult to adapt to socially stressful situations, as food restriction can impact socialisation [26,37,47,98]. In addition, Manti et al. reported that early-treated patients with good metabolic control during childhood were more likely to experience anxiety and depression in adulthood than those with poor metabolic control, leaving the authors to hypothesise that restrictive management strategies may increase psychiatric vulnerability [98].
**Statement #11****Consensus**Despite struggling with long-term adherence to MNT and/or having poor metabolic control, some adults paradoxically have a good self-reported QoL due to becoming accustomed to living with PKU and its management.85%

According to the general population, the Phe-restricted diet and PKU-related symptoms are both associated with disutilities (i.e., decrements in valued QoL) depending on the degree of protein restriction and symptom severity [102]. Unaffected individuals may sometimes more accurately reflect the impact of a disease and its treatment due to adaptation of patients to a particular health state [102]. In PKU, QoL assessments are further complicated by the fact that elevated blood Phe concentrations can lead to clouded judgement, hampering self-evaluation of the impact of PKU and its management on a patient’s QoL [26,103]. Similar to other clinical outcome assessments, generic QoL measurement tools have limited validity in PKU due to the lack of sensitivity to assess the impact of problems specifically encountered by patients with PKU [81,82,103,104]. When applying a PKU-specific QoL questionnaire that considered the burden of diet among other PKU-specific QoL domains, the highest impact scores were indeed related to the emotional impact of PKU and its disease management [81,82]. The QoL of adults with PKU is particularly affected by the continuous worry about blood Phe concentrations, guilt regarding suboptimal adherence to MNT and poor palatability of protein substitutes/medical foods [81,82,105]. It is therefore not surprising that patients with good therapy adherence often perceive their QoL to be better than those who fail to adhere [81,106]. However, strictly adherent patients do not always understand how their lives would benefit from diet normalisation, underestimating the burden of diet. In PKU, gains in QoL domains have been demonstrated by most studies with sapropterin [66,82,107,108]. Compared with non-responsive patients, adults on sapropterin experience fewer mood problems and a lower social burden. These benefits are related to the increase in Phe tolerance, reducing the practical impact of dietary treatment [66,82,107,108]. For pegvaliase, preliminary case reports and expert opinions similarly report improvements in the QoL of adults with PKU related to the ability of patients to consume a more normal diet [109].

### 3.3. Potential Detrimental Health Effects Related to Long-Term Reliance on MNT


**Statement #12**

**Consensus**
The synthetic nature of the Phe-restricted diet can result in micronutrient deficiencies, especially in patients who discontinue protein substitutes/medical foods but continue to restrict their protein intake.92%

Depending on the disease severity, adults with PKU should reduce the intake of natural protein and instead consume synthetic protein substitutes/medical foods (Phe-free amino acids or casein glycomacropeptide-based protein substitutes) supplemented with minerals, vitamins and essential fatty acids to provide sufficient nutrients [4,5]. To compensate for the rapid oxidation of L-amino acids, European and ACMG guidelines recommend that adult patients consume an excess amount of 40% more protein from non-natural sources than the FAO/WHO/UNU safe levels for protein intake or 20–40% more than the dietary reference intake, respectively [4,5]. Because micronutrients play a role in growth, bone health and cognitive functioning, protein substitutes/medical foods should be readily available to all patients, and adherence should be guaranteed through regular dietetic counselling [4,5]. Although the administration of protein substitutes/medical foods should prevent the development of any severe micronutrient deficiencies, studies reported both suboptimal (e.g., choline, potassium, selenium, zinc and essential fatty acids) and excessive levels (e.g., folic acid) of micronutrients in part of the PKU population adherent to MNT [110,111,112,113,114,115]. Because it is unclear if these deviations cause any clinical symptoms, regular monitoring is recommended, and diversity in the composition of different protein substitutes/medical foods should be considered to ensure a balanced diet, irrespective of the disease severity and Phe tolerance [114,116]. In addition, micronutrient deficiencies (e.g., calcium, magnesium, iron, zinc, iodine and vitamin D) are frequently observed in patients on a relaxed diet, including those on pharmacological treatments [117,118]. Most patients on sapropterin reduce the intake of protein substitutes/medical foods while increasing the intake of natural protein. If patients are not well monitored, diet relaxation can cause nutritional inadequacies due to the underuse of protein substitutes/medical foods and development of unhealthy eating patterns [119]. Acquiring new eating habits is particularly difficult for adults with PKU who are accustomed to avoiding natural protein sources throughout childhood, often in favour of foods with high carbohydrate content [120]. Likewise, food neophobia has been reported to occur in some participants receiving pegvaliase [56]
**Statement #13****Consensus**Recent evidence suggests that MNT alters the gut microbiome of adults with PKU, requiring further research to determine its impact on health outcomes.77%

In recent years, interest in studying the gut microbiome of patients with metabolic disorders such as PKU has grown [121,122,123,124]. Although MNT should provide sufficient nutrients to patients with PKU, its composition is not comparable to a normal diet and often results in an increased carbohydrate intake and a higher glycaemic index that can affect the gut microbiota [123]. In addition, the quality of dietary lipids should be considered in future studies. Besides the diet itself, elevated blood Phe concentrations have been hypothesised to affect the gut microbial composition [121]. The cross-talk between the gut and brain is becoming more well established, with disturbances in the communication along the gut–brain axis being correlated with the aetiology of neuropsychological disorders. However, the role of the gut microbiome in PKU remains to be determined [121].
**Statement #14****Consensus**Although future studies are needed, MNT can increase the risk for disordered eating behaviours due to the restrictive nature, limited food choices and constant focus on diet, especially in patients with low Phe tolerance.100%

Abnormal or irregular eating behaviours are inherently related to the restrictive nature of MNT [4,125,126]. As a result, part of the adult PKU population develops disordered eating characterised by feelings of guilt, failure and embarrassment as well as unhealthy food preoccupations and food neophobia [4,56,125]. Similar to the outcomes of QoL assessments, patients with poor metabolic control may be more prone to developing disordered eating than those who are well controlled on MNT [125]. In particular, patients who attempt to adhere to MNT but fail to achieve metabolic control can experience pressure from the metabolic team and treatment environment, triggering disordered eating behaviours and attitudes [125]. However, the lack of PKU-specific eating disorder questionnaires currently limits the identification and assessment of disordered eating behaviours in PKU [100]. When developing these assessments, it should be considered that patients may perceive their restrictive dietary habits as normal (e.g., daily intake of the same food) and that the prevalence of disordered eating in PKU can be underreported due the fact that patients become accustomed to living with unhealthy eating habits. Disordered eating does not meet the criteria for an eating disorder diagnosis (defined as abnormal eating or weight-control behaviours that impair a patient’s physical health or psychosocial functioning) but can—together with Phe-related mental health issues—be a risk factor for developing a clinically diagnosed eating disorder [4,86,125,127]. Hence, patients exhibiting disordered eating patterns should be followed closely by dietitians and psychologists while focusing on achieving blood Phe control and maintaining healthy eating habits regardless of the treatment regimen [4].
**Statement #15****Consensus**Patients with PKU may be at an increased risk of becoming overweight and should be monitored for metabolic comorbidities.100%

Although a recent systematic review and meta-analysis did not find an association between obesity and adherence to MNT in the overall PKU population, a subset of patients with a severe disease phenotype were reported to have a higher body mass index (BMI) than healthy controls [128]. In agreement, some studies suggest that overweight is particularly a problem for uncontrolled patients, showing a correlation between blood Phe concentrations and BMI [129,130,131]. However, when evaluating overweight and obesity in PKU, body composition generates a better picture of a patient’s health than BMI by assessing the proportion of body fat versus lean muscle mass [132,133]. In comparison with an age- and gender-matched control group, Barta et al. demonstrated that body fat in female adult patients with PKU was higher, whereas muscle mass was lower despite the BMI being similar between both groups [134]. These findings were not present in male patients who had better metabolic control, which is surprising because women of childbearing age are often more adherent to treatment to prevent maternal PKU [11]. Generally, there is significant variability between studies in terms of disease phenotypes, age groups, protein restriction and consumption of protein substitutes/medical foods and special manufactured low-protein foods, complicating the comparison of body weight and body composition between PKU individuals and the general population [128]. In addition, not all treatment centres have access to a nutritionist/dietitian, which is one of the reasons for the variability in outcomes between different groups of patients. Irrespective of the aetiology, the overall aim of nutritional management should be the prevention of overweight and obesity, ensuring patients with PKU consume a well-balanced diet. Preventing overweight/obesity will further reduce the risk of developing cardiovascular comorbidities, which are reported to be increased in the adult PKU population [17]. Despite studies showing that blood Phe may independently alter lipid metabolism, cardiovascular risk factors are more likely to be induced by overweight/obesity than by the pathophysiology of the disease itself [129,135,136,137]. Regarding the management of overweight and obesity, it should furthermore be considered that the adult PKU population overall has a sedentary lifestyle, potentially due to the disease burden and related social isolation [131,138,139].
**Statement #16****Consensus ^1^**Adults with PKU are at risk for reduced bone mineral density. The aetiology is multifactorial and may be related to an increase in osteoclastogenesis in response to elevated blood Phe concentrations and/or inadequate intake of nutrients present in protein substitutes/medical foods, particularly in non-adherent patients and those with attempted but unsuccessful adherence.100%^1^ One advisor voted “can’t judge”.

Osteopenia is another important yet controversial health concern in PKU. Similar to studies of obesity, conflicting results have been published on alterations in bone mineral density (BMD) due to variability among the included study participants and assessments used for measuring bone health. A recent systematic review concluded that BMD is reduced in the PKU compared with the general population despite BMD being in the normal range for most patients [140]. In PKU, bone turnover seems to increase with age, but the underlying biological mechanisms remain unclear [140]. According to in vitro studies, blood Phe elevations may increase osteoclastogenesis [141,142,143]. However, evidence in patients with PKU does not consistently support the correlation between blood Phe concentrations and bone impairment, and it is potentially the PKU genotype that may drive the increase seen in bone-related inflammatory cytokines [141,144]. In addition to disease-related factors, the aetiology of reduced bone health is likely multifactorial and should also consider the impact of treatment. For adults on a Phe-restricted diet, protein substitutes/medical foods should provide adequate intakes of nutrients essential for bone health, although reduced BMD has been observed in PKU individuals despite normal levels of calcium, phosphorus and vitamin D [140]. Modan-Moses et al. (2007) suggested that the quality and absorption kinetics of proteins consumed through a normal diet can be different than those derived from synthetic protein sources. However, since this study was published, there have been advancements in the nutritional properties of protein substitutes/medical foods that may overcome these differences [145,146]. In addition, some studies have suggested that the high acid load of protein substitutes/medical foods may increase the urinary extraction of calcium, magnesium and sulphate, which would favour the use of synthetic proteins with a lower renal acid load or glycomacropeptide-based products, although their impact on the bone health of adults with PKU remains to be determined [144,147]. Similar to assessing obesity, it is important to measure the body composition of adults on a protein-restricted diet, as impaired bone health can also be related to deficits in muscle mass [133,147].
**Statement #17****Consensus**For some adults with PKU, protein substitutes/medical foods can cause gastrointestinal discomfort, especially when mitigation strategies are not followed.100%

Comorbidity-claims-based studies reported a significantly increased use of gastrointestinal agents in the adult PKU population compared with matched controls [16,17]. This was confirmed by a patient survey showing that 34% of adult patients experienced digestive problems such as stomach ache and reflux [26]. Despite this, none of the experts reported frequent observations of oesophagitis and gastroesophageal reflux disease in their clinical practice. In case patients do experience gastrointestinal discomfort, the addition of extra water to protein substitutes/medical foods may provide some relief by reducing their osmolality [4].

## 4. Discussion

Despite the availability of pharmacological treatments, MNT remains the mainstay of treatment for many adults with PKU [1]. Theoretically, adherence to MNT should allow all patients to maintain blood Phe concentrations within guideline-recommended target ranges [1,2]. However, nearly all studies in adult PKU populations included a sample of patients with average blood Phe concentrations above 600 µmol/L, with no studies consistently including adult patients with blood Phe concentrations below 360 µmol/L. Based on this lack of evidence and practice experience, it was the opinion of the expert panel that the effectiveness of MNT is limited for adults in the long term and usually only allows patients with a milder disease phenotype who tolerate more protein to maintain lifelong blood Phe control. In addition, there was agreement among the experts that even if patients strictly adhere to MNT, the majority cannot sustain blood Phe concentrations within the physiological range. Although there is no evidence to support this claim, so far, not a single study has compared the outcomes of adult patients with physiological Phe versus those with higher blood Phe, suggesting only few are able to achieve these levels through adherence to MNT. It should furthermore be noted that some studies classified patients as being either adherent or poorly adherent to MNT based on their blood Phe concentration without assessing their actual natural protein intake [81]. Although this assumption may be true for most adults with PKU who tend to relax their diet in childhood, some adult patients do not seem to be able to achieve metabolic control despite adhering to the dietary restrictions. Nevertheless, the reality remains that many adult patients are unable to strictly adhere to MNT not only due to the burden of treatment but also because in some countries, the availability of protein substitutes/medical foods and adequate access to adult services and dietetic care remain a longstanding challenge in the management of PKU.

Due to the lack of published evidence, there remains uncertainty on the safety of current guideline-recommended target ranges. Although childhood Phe remains the most important determinant of early and late neurocognitive outcomes, the limited effectiveness of MNT in the long term can still increase the risk for developing deficits in neurocognitive functioning in adulthood [15,37,39,41,42,46,47,148,149]. Even when controlling for historic Phe levels, adults were reported to experience cognitive difficulties in executive functioning, processing speed, motor skills and visuospatial skills, of which the severity correlated with concurrent blood Phe concentrations [37,148,150]. There is therefore no debate on the need for lifelong treatment to prevent these PKU-related cognitive symptoms. However, guidelines currently disagree on the safety of blood Phe target ranges in adulthood due to the paucity of data on PKU adults sustaining their blood Phe within recommended ranges [1,2]. In addition, the entire expert panel agreed that despite metabolic control, subtle neurocognitive deficits may still be present in early-treated adult patients due to current guideline-recommended treatment targets being higher than the physiological blood Phe range [1,2]. However, until more studies can include adults with PKU who are able to achieve physiological Phe, this claim will remain largely speculative. Hence, for now, the importance of lifelong adherence should be emphasised to patients relying on MNT, allowing the majority of adults with PKU to obtain good QoL and other psychosocial outcomes while preventing the most severe PKU-related symptoms [37]. Nevertheless, there was agreement among the expert panel to offer adults with PKU more effective pharmacological treatments when available. The benefit/cost ratio of these treatments will depend on the benefits that can be accomplished by maintaining lower blood Phe concentrations and their capacity to improve patient-reported outcomes, which can be compromised by the burden of the current standard of care [102]. If proven that substantial benefits can be gained with lower blood Phe concentrations, current treatment ranges should be reconsidered in the future.

It has been suggested that both elevated blood Phe concentrations and severe protein restriction together with the intake of synthetic protein substitutes/medical foods may also compromise a patient’s physical health [29]. Many somatic comorbidities have been identified in retrospective claims-based studies, out of which the expert panel considered overweight/obesity, osteopenia and gastrointestinal issues to be potentially associated with long-term reliance on MNT [16,17]. A similar observation was made in a recent review in describing the possible link between protein restriction and physical health manifestations in PKU [29]. However, as for all PKU-associated comorbidities, it remains impossible to determine whether or not these relate to blood Phe, the restrictive nature of MNT as the primary intervention, suboptimal adherence to MNT or any other yet-to-be-identified pathophysiological mechanism. Therefore, the expert panel strongly recommended that patients maintain a balanced diet with adequate nutrition support, preventing any micronutrient deficiencies and detrimental health effects that can be related to consuming an unhealthy diet. In the retrospective claims-based studies, renal insufficiency was also hypothesised to be related to the consumption of protein substitutes/medical foods [16,17,151]. However, renal diseases are generally not experienced by children with PKU, who derive most of their protein intake from synthetic amino acids [17]. Hence, the expert panel did not endorse the statement on the association between renal insufficiency and long-term reliance on MNT and does not regularly assess renal function in adults with PKU.

In addition to physical health manifestations, the US retrospective claims-based study specifically evaluated the prevalence of neuropsychiatric comorbidities in adults with PKU, out of which many occurred more frequently in the PKU population than the general population [86]. It is well established that elevated blood Phe concentrations contribute to a great extent to the development of neuropsychiatric and mental health problems, although the expert panel agreed that the burden of MNT can exacerbate these issues. Because most early-treated adult patients have blood Phe concentrations either above 360 or 600 µmol/L, current studies cannot differentiate the impact of elevated blood Phe from the burden of dietary treatment. As a result, both aspects should be considered when managing patients on MNT who present with any neuropsychological or psychosocial PKU-associated comorbidities, such as anxiety, stress and depression. According to the experts, the only neuropsychiatric comorbidity that can inherently be related to MNT rather than blood Phe is disordered eating due to the overarching effect food can have on the life of a patient with PKU. Together with the relatively high prevalence of mental health disorders (e.g., anxiety), these disordered eating behaviours can contribute to development of eating disorders in the adult PKU population [86]. Similar conclusions were reported by Burton et al. (2022), who proposed a list of questions to screen for disordered eating patterns in PKU [100]. Initially, two statements on disordered eating were included in this study, but after feedback and revisions, these were combined into one statement. Together with the removal of one statement on renal insufficiency, a total of 17 statements on the limitations of the long-term effectiveness of MNT and its impact on the outcomes of adults with PKU were endorsed by the expert panel through a modified Delphi process.

## 5. Limitations

There remains much uncertainty on the long-term outcomes of adults with PKU as well as a general lack of evidence to support the safety of guideline-recommended target ranges in adulthood. Therefore, some of the statements, especially those that describe the limited long-term effectiveness of MNT, are solely based on the opinion of the expert panel (although we used a modified Delphi process to achieve consensus based on clinical practice experience). Because most of the experts are practicing in countries where newborn screening programmes for PKU have been implemented for more than 50 years, the statements focus on early-diagnosed and -treated adults with PKU. Nevertheless, similar opinions may be true for late-diagnosed patients, who are generally even more vulnerable to the long-term detrimental effects of elevated blood Phe. In addition, the current findings cannot be extrapolated to the paediatric PKU population, who may have different unmet needs.

## 6. Conclusions

There remains an unmet treatment need in the adult PKU population that was reflected through 17 statements that describe the limitations of MNT with regards to the limited long-term effectiveness of MNT, the burden of long-term reliance on MNT and the potential detrimental health effects related to long-term reliance on MNT. Globally, MNT is the mainstay of PKU treatment. However, due to the burden of MNT in the long term, many adult patients are unable to follow it adequately and therefore may be at risk for detrimental health effects that can be related to the lack of metabolic control and/or micronutrient deficiencies. Future research with existing, emerging and more effective treatments should focus on the identification and assessment of patient-reported outcomes to clearly differentiate the impact of blood Phe from the burden of treatment, evaluate whether alternative treatments can address the unmet needs of the adult PKU population and determine if patients would benefit from achieving physiological Phe.

## Data Availability

No new data were created or analysed in this study. Data sharing is not applicable to this article.
